# A Recurrent Cervical Neurenteric Cyst Treated Anteriorly: Safe, Gross-Total Excision Facilitated by Prophylactic Unilateral Vertebral Artery Exposure, Microdissection, and Spinal Cord Monitoring—A Case Report and Technical Note

**DOI:** 10.1155/2018/7620182

**Published:** 2018-03-04

**Authors:** Kazunobu Kida, Toshikazu Tani, Tateo Kawazoe, Makoto Hiroi

**Affiliations:** ^1^Department of Orthopaedic Surgery, Kubokawa Hospital, 902-1 Mitsuke, Shimanto-cho, Takaoka-gun, Kochi 786-0002, Japan; ^2^Laboratory of Diagnostic Pathology, Kochi Medical School, Kohasu Oko-cho, Nankoku, Kochi 783-8505, Japan

## Abstract

This study reports on a 67-year-old woman with partial Brown-Séquard syndrome due to a recurrent cervical neurenteric cyst at C3 to C4. The myelopathic symptoms reappeared 22 years after a previous shunting operation performed posteriorly with a silicone tube connecting the intradural cervical cyst cavity to the subarachnoid space. We have now succeeded in removing the cyst nearly completely with the anterior approach. The surgical procedure consisted of right vertebral artery exposure at C3 and C4 and a subtotal corpectomy of C3 followed by microdissection of the cyst, duraplasty, and iliac strut graft fusion. Spinal cord monitoring with motor-evoked potential studies helped us safely dissect the cyst wall tightly adhering to the spinal cord. Duraplasty with Gore-Tex patch-grafting in conjunction with postoperative lumbar subarachnoid drainage worked well in preventing a spinal fluid fistula. At two years after surgery, the patient showed a nearly complete return of function without any recurrence of the cyst.

## 1. Introduction

Spinal neurenteric cysts (SNCs) are relatively rare congenital lesions. The cysts tend to be located ventrally to the spinal cord in the cervical and thoracic regions [1]. Surgical approach to these lesions can be achieved either anteriorly or posteriorly [2]. The posterior approach has been preferred in many cases reported, although the anterior approach allows for a nearly complete and safe excision of the cyst with minimal manipulation to the spinal cord [3]. According to a few recent reviews of postsurgical outcome analyses of this condition, incomplete surgical excision posteriorly carries a higher cumulative risk of recurrence as the follow-up period increases. Despite these data, only a single report to date has presented a case of a delayed recurrence of the SNC after partial excision posteriorly, which was addressed with anterior revision surgery [4]. We report on a late recurrence case of cervical SNC successfully treated with the anterior approach for partial Brown-Séquard syndrome, developing 22 years after a marked shrinkage of the cyst and complete return of function with posterior shunting operation. This report describes preoperative evaluations, detailed operative techniques, and postoperative management with emphasis on surgical strategies for safe, complete excision of the SNC involving extensive foraminal extension and tight adhesion to the spinal cord.

## 2. Case Report

### 2.1. Clinical Picture

A 67-year-old woman presented with the chief complaint of numbness and clumsiness in the right hand lasting 7 months. She managed to write but not functionally and could walk unaided but with some difficulty. Physical examination revealed partial Brown-Séquard syndrome with motor deficit, which predominantly affected the right upper limb and sensory impairment by a pinprick in the left upper and lower limbs. Muscle stretch reflexes, while generally responding normally, showed a hyperactive biceps reflex and a diminished ankle jerk bilaterally. What is noteworthy about the history of this case is that she had a shunting operation for the intradural cervical cyst using a silicone tube to create a communication between the cyst cavity and the subarachnoid space with a posterior approach 22 years ago. She recovered completely after the operation, without displaying any recurrence of symptoms originating from the cervical lesion for a period of more than 20 years.

### 2.2. Radiologic Findings

**Figure 1:**
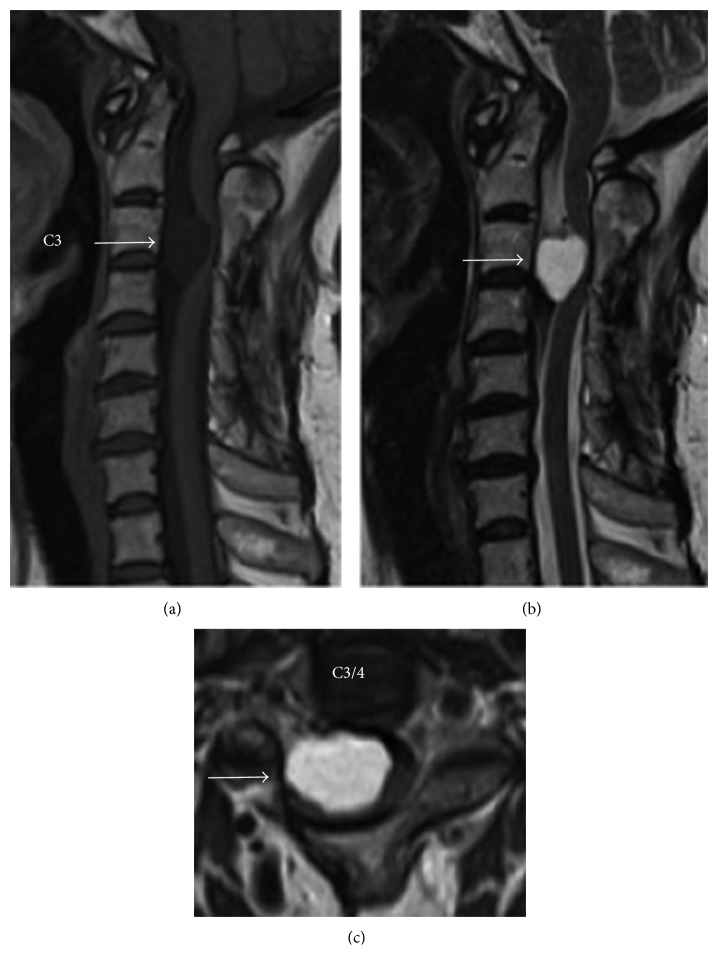
Magnetic resonance imaging (MRI) showing the intradural extramedullary cystic mass lesion. The spinal cord was completely stretched out over this ventral cyst (arrows). (a) T1-weighted sagittal image. (b) T2-weighted sagittal image. (c) T2-weighted axial image.

**Figure 2:**
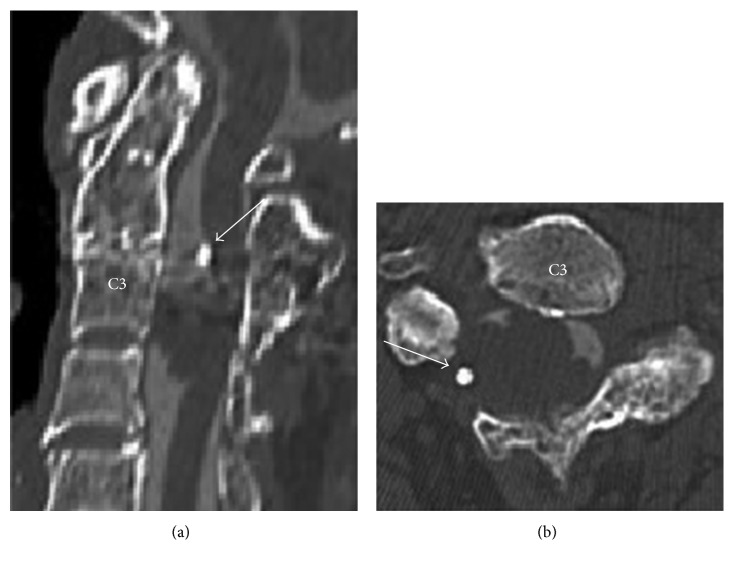
Computed tomographic myelography also showing clearly delineated intradural extramedullary mass and the previously placed shunting tube (arrows). (a) Sagittal image. (b) Axial image.

MRI of the cervical spine revealed an intradural extramedullary cystic mass lesion ventrally to the spinal cord at the C3 to C4 level with the spinal cord tightly stretched out over this ventral cyst (Figure 1). The content of the cyst fluid appeared slightly hyperintense compared to cerebrospinal fluid (CSF) on both T1- and T2-weighted MRIs. T1-weighted MRI sequences after gadolinium administration showed no enhancement of the cyst wall. Computed tomographic myelography also showed clearly delineated intradural extramedullary mass with the previously placed shunting tube (Figure 2). An isolated bony union between C2 and C3 found on the plain radiograph proved to be an unintended fusion, which had taken place after the first shunting operation using expansive open-door laminoplasty. Based on these radiological findings consistent with the diagnosis of SNC, we opted to perform surgery from the front.

### 2.3. Lumbar Subarachnoid Drain Placement

Following preoperative general anesthesia, we inserted a lumbar spinal drain percutaneously into the subarachnoid space to prepare for continuous CSF drainage after surgery involving durotomy [5]. The drain tube remained clamped during surgery.

### 2.4. Spinal Cord Monitoring

Intraoperative spinal cord monitoring consisted of stimulating the brain transcranially with corkscrew-type needle electrodes placed into the scalp 2 cm anteriorly and 5 cm laterally to the vertex on both sides and recording motor-evoked potentials (MEPs) from the bilateral tibialis anterior and the abductor hallucis with two monopolar needle electrodes inserted intramuscularly 2–4 cm apart [6]. A train of 4 TES pulses with an interstimulus interval of 2 ms delivered after propofol-opioid total intravenous anesthesia successfully evoked baseline MEPs in those muscles.

### 2.5. Surgery

**Figure 3:**
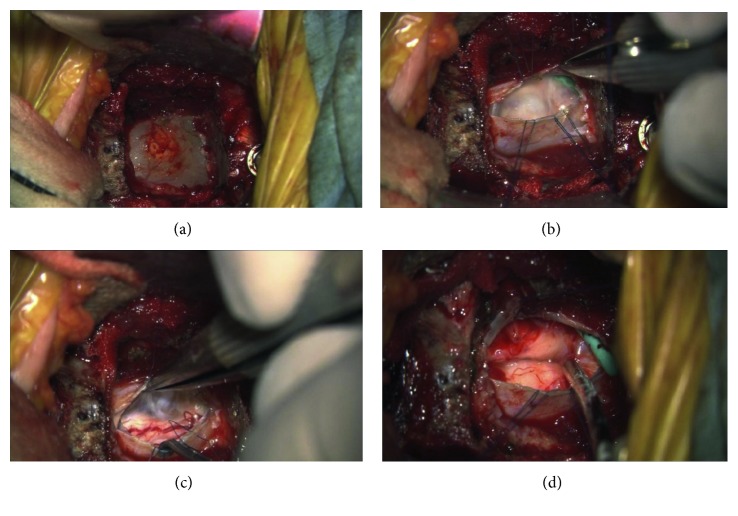
Intraoperative microscopic view. (a) Expanded and thinned dura. (b) White and glossy inner side and the remaining shunt tube. (c) Cyst wall densely adhering to the surface of the spinal cord. (d) After radical resection of cyst wall.

A transverse incision on the right side was made at the level of the hyoid bone from just across midline to the sternocleidomastoid muscle laterally. After exposure of the anterior aspect of the vertebral bodies of C2, C3, and C4 with a standard approach, bilateral longus colli muscles were elevated from the vertebral bodies laterally, and the right transverse processes of C3 and C4 were exposed. By removing the anterior tubercles of the two transverse processes with a Kerrison rongeur, the vertebral artery was unroofed anteriorly to protect the artery during dissection of the cyst located with more right-sided extension. After the C3/4 disc was incised and removed, subtotal corpectomy of C3 and resection of one-third of the rostral portion of C4 vertebral body were carried out. Under the operative microscope, all bony cortexes were removed with a high-speed diamond-tipped burr, fully exposing the posterior longitudinal ligament (PLL). The uncovertebral joint was identified laterally on both sides, and right uncovertebral joint was resected completely with great care to avoid damage to the vertebral artery. The PLL was then removed cautiously from these areas, and the dura was revealed clearly. Epidural bleeding from venous plexus was controlled by bipolar electrocautery and a hemostatic agent. The dura was expanded and thin so that we could locate the exact position of the cyst through the dura without ultrasonic confirmation (Figure 3(a)). A slightly right-sided longitudinal dura incision was chosen for the location of the cyst. After the incision, because both the arachnoid membrane and the cyst wall were densely adhering to the inner dura layer, CSF and the cyst content leaked out unexpectedly. A white and glossy inner side of the cyst could be seen, through which the surface of the spinal cord was visualized. The shunt tube placed during the first surgical operation was observed in the cyst (Figure 3(b)). We left the tube in place without trying to extract it, thereby reducing the chance of damage to vascular, meningeal, or neural structures potentially adherent to the tube. We tried to remove the entire cyst wall from surrounding structures, but it could not be dissected off the spinal cord surface completely (Figure 3(c)). One portion of both the dura and the arachnoid membrane, where the cyst wall could not be removed radically, was resected. The dura was closed with patch-grafting using a Gore-Tex, the expanded polytetrafluoroethylene (ePTEE) sheet, followed by augmentation with fibrin adhesive (Beriplast P). A tricortical iliac crest strut was harvested and then trimmed and shaped to match the vertebral recipient site. Under the gentle manual traction of the head, the graft was tamped into position. The subfascial drain was placed with very low pressure, and the wound was closed in layers. MEPs for spinal cord monitoring showed no significant amplitude reduction throughout surgery.

### 2.6. Postoperative Course

**Figure 4:**
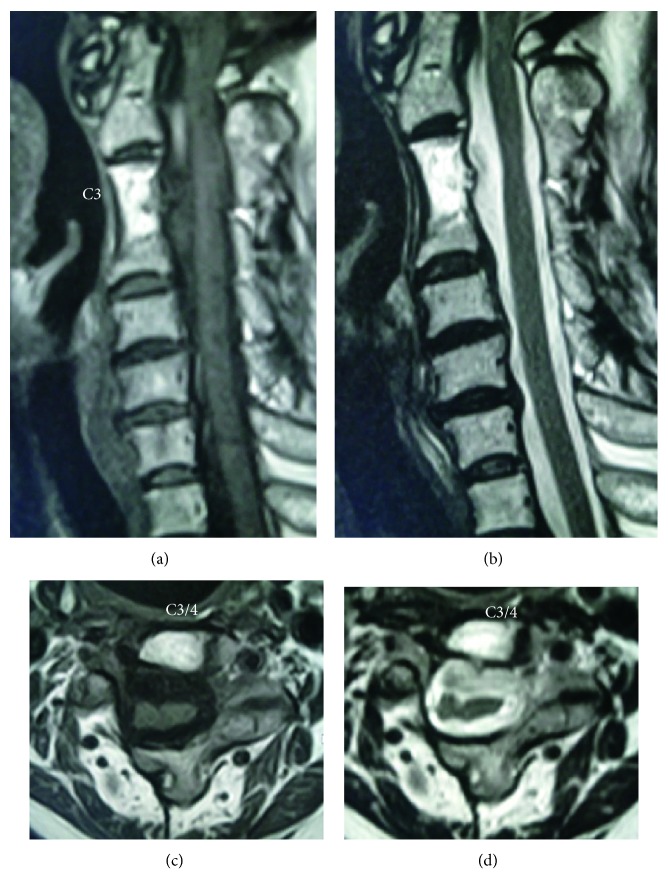
MRI showing neither recurrence of the cyst nor leakage of the cerebrospinal fluid at 24 months after surgery. (a) T1-weighted sagittal image. (b) T2-weighted sagittal image. (c) T1-weighted axial image. (d) T2-weighted axial image.

Lumbar subarachnoid drainage of CSF was started immediately following surgery and continued until day 5 after surgery, with the patient kept in bed resting. The drainage with an average daily drain output of 200 ml worked well in preventing spinal fluid leak through the operative wound. On day 12 after surgery, the patient was allowed to ambulate with a hard cervical collar, which was kept in place for 3 months. The patient showed a progressive recovery of the myelopathic symptoms and eventually regained normal function at the 2-year visit, when she had a solid fusion on radiographs and showed neither signs of a cyst recurrence nor a CSF fistula on MRIs (Figure 4).

### 2.7. Histopathology

**Figure 5:**
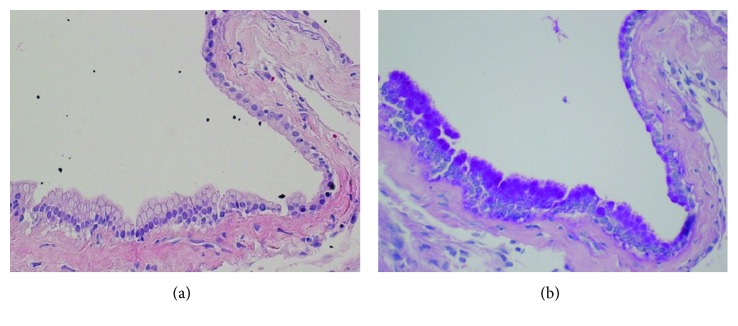
Histopathological examination. (a) Cystic wall lined by a cuboidal to columnar epithelial cells (original magnification ×400, H&E). (b) Those cells were periodic acid-Schiff (PAS) reaction positive (original magnification ×400).

Histopathological examination of the cyst demonstrated a cystic wall lined by cuboidal to columnar epithelial cells and those cells were periodic acid-Schiff (PAS) positive, all consistent with the diagnosis of SNC. According to Wilkins and Odom's histopathological classification [7], this cyst is classified as type A (Figure 5).

## 3. Discussion

SNCs, rare congenital lesions [8], may result from inappropriate separation of the embryonic notochordal plate and presumptive endoderm during the third week of embryogenesis, as generally believed [9]. Consistent with their congenital origin, the cystic lesions tend to be associated with various bony anomalies (approximately 50% of prevalence) including fused vertebrae and Klippel–Feil syndrome [10], but those which develop in the elderly population, those located in the cervical spine, and those histologically classified as type A do not [3, 11]. In fact, an isolated bony union between C2 and C3, found in our patient aged 67 years with a SNC classified as type A, proved not to be congenital in origin but an unintended fusion caused by a previous shunting operation.

As reported previously [1, 8, 9], approximately 90% of SNCs are intradural extramedullary in location and fewer than 5% intramedullary. The majority of these cysts are located ventrally to the spinal cord with approximately 50% of them found in the cervical spine. The cervicothoracic region is the second most common site of involvement. These predilections for location of SNCs are consistent with the MRI findings seen in our case. A high protein content and/or fat-like substance commonly identified in the cyst contents [12] can explain a slightly higher signal intensity of the current cyst fluid than that of CSF on both T1- and T2-weighted MRIs. Also consistent with an imaging characteristic of SNCs, contrast-enhanced MRIs showed a complete lack of enhancement, indicating a thin-walled cyst.

According to previous reports, surgical intervention for SNCs has been mostly approached posteriorly with laminectomy or laminoplasty despite their ventral location to the spinal cord [13]. In fact, our patient previously underwent a shunting operation using a silicone tube to establish a communication between the cyst cavity and the subarachnoid space with a posterior approach 22 years ago, which eventually failed to function. Osenbach et al. also reported a shunting operation with stent catheters, instead of a silicone tube, placed between the cyst cavity and the subarachnoid space, possibly as a good option to prevent the fluid pathway from closing with scarring [14]. Some authors, however, contend that an anterior approach, if feasible, serves better than a posterior approach for removing the ventral cystic lesions completely to reduce the chance of recurrence of the cysts [15]. The same consideration applies to our cystic lesion with a purely ventral location to the spinal cord. More right-sided extension of the cyst explained a partial Brown-Séquard pattern of the patient's neurologic deficits. Notably, our patient had previously undergone a shunting operation posteriorly, which, due to postoperative epidural and/or intradural scarring and adhesion, may make it more difficult to posteriorly dissect recurrent cysts without overmanipulation of the spinal cord [16]. Considering these factors together with the patient's age and subaxial location of the cyst, we chose an anterior approach. Our surgical strategy consisted of a standard anterior cervical approach, partial exposure of the vertebral artery unilaterally, a subtotal corpectomy, durotomy, microdissection of the cyst, duraplasty, and a two-level fusion with an iliac crest strut graft.

Some procedures deserve special mention as follows: (1) More right-sided extension of the cyst required the exposure of the right vertebral artery at C3 to C4 by removing the anterior tubercles of the two transverse processes, which facilitated a safe, complete removal of the lesion. (2) Microdissection failed to completely separate the anterior wall of the cyst from the dura, resulting in partial resection of the dura inseparable from the cyst wall followed by duraplasty to restore the CSF flow and prevent intradural scarring. The duraplasty using Gore-Tex patch-grafting of the dural defect in conjunction with postoperative lumbar subarachnoid drainage helped avoid CSF leak through the operative wound, thereby preventing CSF fistula. (3) Microdissection between the posterior wall of the cyst and the spinal cord surface failed to completely dissect off the cyst wall from its bed, even under high magnification, because of the tight adhesion. We managed to remove nearly the entire cyst wall, but a very small portion was left to avoid spinal cord compromise (Figure 3(d)). This critical stage of dissection of the cyst indicated a relative advantage of the anterior, as compared with the posterior approach, for this condition because if we had chosen the posterior approach, the dissection would have been more difficult and hazardous, probably leading to a larger portion of the cyst wall densely adhering to the spinal cord left unremoved. (4) Intraoperative spinal cord monitoring using MEP, although requiring trains of stimuli after intravenous anesthesia with propofol-opioid, worked well in preventing iatrogenic neurologic complications.

With an anterior approach, one can directly access SNCs located ventrally to the spinal cord. Selective use of a series of anterior procedures described here, based on proper diagnosis and surgical planning, could help us safely and nearly completely remove SNCs without complications.

## 4. Conclusion

A recurrent SNC with myelopathy presented us with the technical challenge of safely removing the lesion in its near entirety. Although previous reports mostly employed a posterior approach for the initial surgical intervention for SNCs, the anterior strategy served better for microdissection between the spinal cord and the cyst wall under MEP monitoring, as indicated in this study. Partial exposure of the right vertebral artery necessary for covering MRI evidence of more right-sided cystic extension allowed for its adequate removal. Duraplasty in conjunction with postoperative lumbar subarachnoid drainage helped prevent spinal CSF fistula.
